# Diagnostics and Therapeutics in Ophthalmology

**DOI:** 10.3390/jpm15010028

**Published:** 2025-01-14

**Authors:** Andreas Arnold-Vangsted, Yousif Subhi

**Affiliations:** 1 Department of Ophthalmology, Rigshospitalet, 2100 Copenhagen, Denmark; andreas.arnold-vangsted@regionh.dk; 2 Department of Clinical Medicine, University of Copenhagen, 1172 Copenhagen, Denmark; 3 Department of Clinical Research, University of Southern Denmark, 5230 Odense, Denmark

Clinical research aims to answer questions that are of importance to daily clinical practice in order to improve and optimize disease diagnosis and therapy, which ultimately impacts patients’ well-being. From a larger perspective, good clinical research may improve healthcare organization and cost-effectiveness [1]. In that regard, one cannot underestimate the importance of focusing on clinical research in diagnostics and therapeutics, in relation to ophthalmology.

Globally, it is estimated that 33.6 million individuals suffer from blindness and that 206 million individuals suffer from moderate to severe vision impairment [2]. A substantial number of these cases are potentially avoidable [2]. In other cases, improvements in their clinical management may strongly benefit patients. Even small improvements in clinical management may mean the world to the patients [3,4].

This Editorial discusses the Special Issue “Diagnostics and Therapeutics in Ophthalmology”, published in the *Journal of Personalized Medicine*. Nineteen manuscripts were submitted, which all underwent rigorous peer-review processes. Finally, 13 of these manuscripts were accepted for publication. It is important to acknowledge that while good clinical research can improve clinical outcomes and benefit the patients, poor studies may mislead and even potentially harm the patients [5,6].

Three studies dealt with myopia, a condition with an increasing prevalence, particularly in Asia [7], in which exciting treatments are emerging. Hvid-Hansen et al. (Contribution 1) investigated the reproducibility of pupil size measurements in myopic children. In another study, Hvid-Hansen et al. (Contribution 2) reported the results of a six-month-long randomized clinical trial on atropine eye drops for combatting myopia progression in children. A follow-up study by Hansen et al. (Contribution 3) reported the two-year results of safety and efficacy.

One study focused on the anterior part of the eye. Azizi et al. (Contribution 4) systematically reviewed the surgical treatment options of corneal shield ulcers in vernal keratoconjunctivitis.

Two studies dealt with glaucoma. Świerczyńska et al. (Contribution 5) presented a very extensive deep phenotyping of a case with Aicardi–Goutières syndrome, which presented with ophthalmic symptoms at a very early age. Tanito (Contribution 6) presented a study with large-scale data from Japan and gave excellent insights into the nationwide trends in glaucoma surgeries.

Vascular diseases of the retina are prevalent and constitute an important number of patients in eye clinics [8]. Five studies dealt with various aspects of this topic. Cehofski et al. (Contribution 7) analyzed protein changes in pigs receiving bevacizumab for retinal vein occlusion and identified that 59 proteins were subject to changes following drug administration. Ørskov et al. (Contribution 8) calculated the validity parameters of retinal artery occlusion in nationwide databases in Denmark and found acceptable levels for scientific purposes. Ba-Ali et al. (Contribution 9) found that obstructive sleep apnea was associated with a lower risk of vision-threatening diabetic retinopathy. Nissen et al. (Contribution 10) reported on the performance of a support vector machine learning software to diagnose diabetic retinopathy. Subhi et al. (Contribution 11) reported on the incidence, burden of therapy, and forecasts of the anti-VEGF treatment of diabetic macular edema in Denmark.

One study focused on diseases of the choroid. Central serous chorioretinopathy is a topic with a lot of controversies [9], and it is imperative that evidence synthesis is based on an extensive and sufficient literature evaluation. In that regard, Boberg-Ans et al. (Contribution 12) evaluated the scientific literature database coverage of randomized clinical trials for central serous chorioretinopathy.

One study dealt with retinal detachment, which is an emergency that can lead to blindness. Aykut et al. (Contribution 13) gave insight into a useful method for pneumatic retinopexy that can be used for rhegmatogenous retinal detachment.

Taken together, these studies deal with the important topic of our clinical practice as ophthalmologists.
